# Frailty and functional recovery after cardiac surgery: a randomized pilot trial of extended exercise-based rehabilitation

**DOI:** 10.1186/s12877-026-07192-5

**Published:** 2026-02-24

**Authors:** Egle Tamuleviciute -Prasciene, Kristina Balne, Inesa Kuznecova, Aurelija Beigiene, Vitalija Stonkuviene, Raimondas Kubilius

**Affiliations:** 1 Department of Rehabilitation, Lithuanian University of Health Sciences, Eiveniu Str. 2, Kaunas, LT-50161 Lithuania; 2 Department of Cardiology, Lithuanian University of Health Sciences, Eiveniu Str.. 2, Kaunas, LT-50161 Lithuania

**Keywords:** cardiac rehabilitation, cardiac surgery, frailty, Edmonton frailty scale, exercise training

## Abstract

**Objectives:**

Pilot branch study of the *FrailHeart* clinical trial aimed to assess the feasibility and preliminary performance of the Edmonton Frail Scale (EFS) for detecting changes in frailty status over a 3-month follow-up period after cardiac surgery in older adults, and to explore frailty within the context of exercise-training–based (ET) cardiac rehabilitation (CR) in order to inform the design and implementation of future definitive studies targeting frailty in this population.

**Methods:**

Patients who arrived at an inpatient CR hospital after open-heart surgery between November 19, 2020, and January 3, 2022, were invited to participate. Out of 336 assessed for eligibility, 100 patients (38 females, 62 males) met the inclusion criteria and were randomized into intervention (IG, *N* = 50) and control groups (CG, *N* = 50). All participants underwent comprehensive inpatient CR based on ET. After discharge, IG continued a 12-week home ET program while CG maintained usual physical activity. Assessment times: before randomization (admittance to CR) (V1); CR completion (V2), and three months post-CR (V3). Assessment included clinical examination: six-minute walk test (6MWT), veloergometry (peak workload (W), maximal heart rate (max.HR, beats/minute), metabolic equivalent (MET), timed up and go (TUG), frailty (EFS score/status).

**Results:**

As a result of inpatient-CR all measured parameters except max. HR enhanced significantly for all study participants (6MWT, TUG, EFS, W, MET (p < 0.001)). Analysis of differences between groups at V2 showed significantly better physical capacity for IG (MET, p = 0.037), without statistical significant differences in other outcomes. 58 patients attended V3 and completed the entire study (IG n = 33, CG n = 25). Only W, MET and EFS enhanced for all patients significantly (p < 0,01) without statistical differences between groups. Changes in frailty status over time (V1-V3) were statistically significant (V1-V2: IG p < 0.01; V1-V3: CG p < 0.05; V1-V3: IG p < 0.05) whereas no statistically significant between-group differences were observed (χ² p = 0.32). All IG patients were able to successfully participate in the home ET program, no AE were registered.

**Conclusion:**

Within-group analyses indicated that EFS scores changed over time, suggesting that the instrument is capable of capturing short-term variation in frailty status in this clinical context. The structured CR ET program was found to be feasible and safe in this high-risk population. Although reductions in frailty scores were observed over time within groups, between-group differences were not consistently demonstrated, and the study was not powered to establish intervention effectiveness. Future adequately powered, multi-center studies incorporating comparator instruments are needed to confirm the responsiveness of the EFS and to clarify the effects of CR and ET on frailty trajectories in this population.ClinicalTrials.gov (No. NCT04636970).

**Trial registration:**

This study was registered at ClinicalTrials.gov (ID NCT04636970). First Posted 19/11/2020.

## Introduction

 Frailty is a multidimensional syndrome characterizing diminished physiological reserves and increased vulnerability to adverse endpoint [1]. Contemporary preoperative frailty assessment has become a major component of clinical decision making, particularly in aging populations. Frailty measurement is an ongoing topic in recent literature; analysis of other publications showed that there are at least seven different validated scales for frailty evaluation, including the Fried Frailty Phenotype, the Edmonton Frailty Scale (EFS), the Clinical Frailty Scale (CFS), the Frailty Index (FI), the FRAIL scale, the Tilburg Frailty Indicator (TFI), and the Gérontopôle Frailty Screening Tool (GFST) [2]. While the Fried criteria primarily focus on physical frailty by assessing five components (unintentional weight loss, exhaustion, low physical activity, slowness, and weakness), other tools, such as the EFS, incorporate multidimensional aspects, including cognitive, psychological, and social domains. Although the Fried Frailty Phenotype (FFP) is widely regarded as a fundamental and validated tool for assessing physical frailty, recent literature emphasizes that no single instrument serves as a universal “gold standard.” As highlighted by Deng and Sato (2024) [3] the Fried criteria remain a key method—especially for identifying physical components such as muscle weakness and reduced endurance—EFS offers a more comprehensive evaluation by encompassing cognitive, psychosocial, and functional domains. The association between frailty and surgical outcomes is of growing interest, with evidence that frailty is an independent predictor of increased postoperative morbidity, more adverse long-term outcomes, and delayed recovery of patients undergoing cardiac surgery [4, 5]. Originally developed for geriatric populations, EFS demonstrated its prognostic value in patients undergoing cardiac procedures such as transcatheter aortic valve implantation [6] and coronary artery bypass grafting [7, 8]. The EFS is notable for its ease of use and comprehensive assessment framework, covering various aspects of patient health as in cardiac surgery patients frailty most frequently results from a combination of elderly age, comorbidities, and the physiological stress of surgery and requires strategies customized for the optimization of recovery [9, 10].

There is evidence that structured and supervised exercise training (ET) programs could decrease frailty by enhancing muscle strength and aerobic capacity along with general functional status [10, 11]. While cardiac rehabilitation (CR) is recognized as an important part of recovery after cardiac surgery, additional studies further validate the cardioprotective effects of CR in modifying frailty and improving clinical outcomes. Exercise training in the context of rehabilitation programs has been shown to improve frailty scores, functional capacity, and quality of life after cardiac surgery [11, 12], and recent evidence further reflects the positive influence of physical activity in other population groups [12, 13]. Integrating frailty assessments using tools such as the EFS alongside individualized rehabilitation regimens may play a larger role in improving recovery and optimizing patients’ overall quality of life following cardiac surgery [11, 14–16].

This pilot branch study of the *FrailHeart* clinical trial aimed to assess the feasibility and preliminary performance of the Edmonton Frail Scale (EFS) for detecting changes in frailty status over a 3-month follow-up period after cardiac surgery in older adults, and to explore frailty within the context of exercise-training–based cardiac rehabilitation in order to inform the design and implementation of future definitive studies targeting frailty in this population.

## Materials and methods

### Study population

**Fig. 1:**
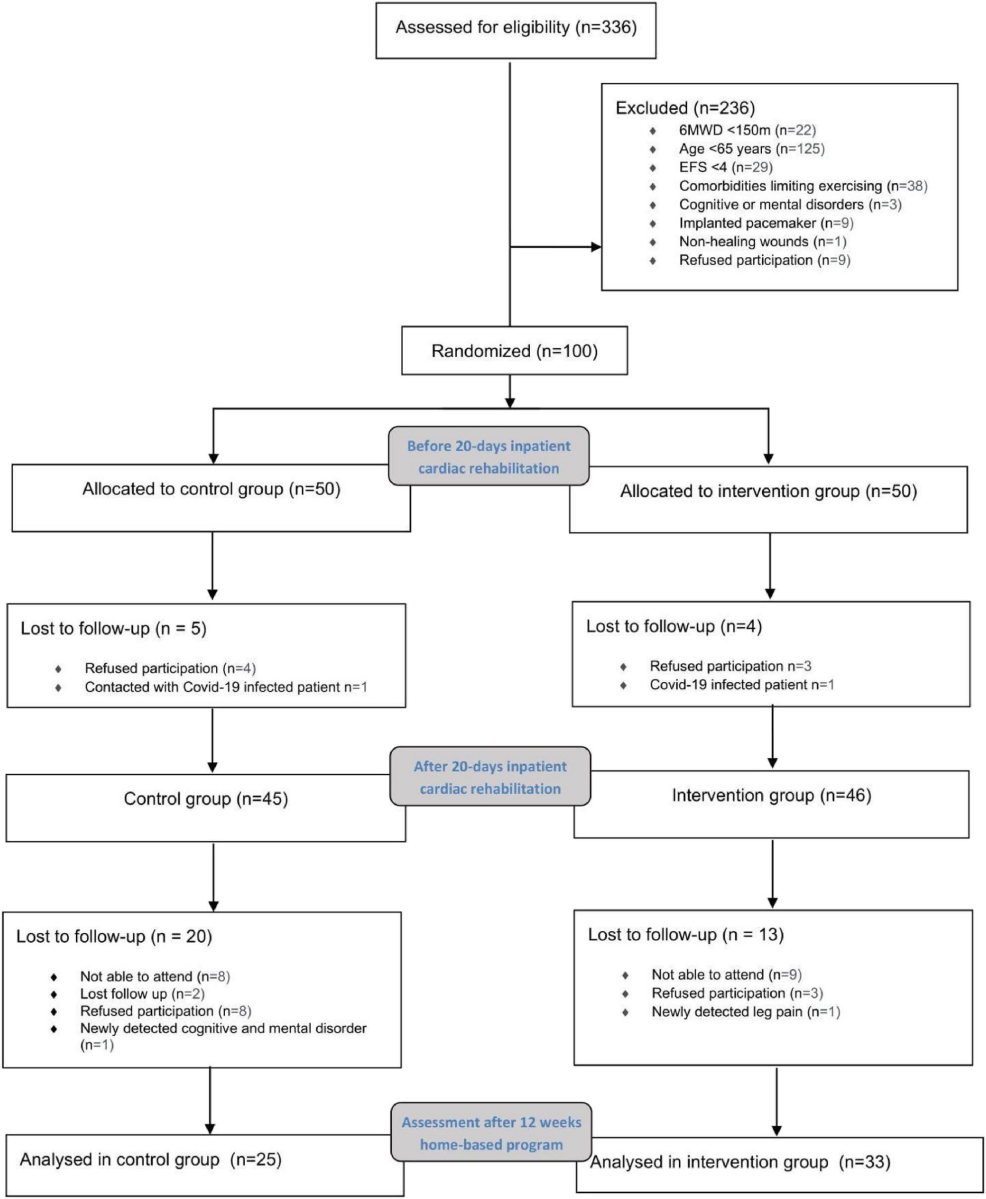
Patient enrollment flow scheme. Here *N* denotes the number of patients

The patients after open-heart surgery, who arrived at Kulautuva Rehabilitation Hospital of Kaunas Clinics (Kulautuva, Lithuania) from 19th of November 2020 till 3rd of January 2022, were invited to participate in the study (Fig. 1). Assigned written informed consent according to the principles expressed in the Declaration of Helsinki was obtained from all eligible patients before participation. The study was approved by the Kaunas Region Biomedical Research Ethics Committee (No. BE-2-99). The study is described in detail in ClinicalTrials.gov (No. NCT04636970). The study was conducted and reported in accordance with the CONSORT guidelines for randomized controlled trials. Patients were assessed for eligibility on the first day of admittance to the rehabilitation hospital according to inclusion and exclusion criteria. (Table 1). Both IG and CG participated in comprehensive CR based on ET during inpatient rehabilitation and received the usual care. The CG was asked to maintain their usual physical activity, while patients randomized to IG after discharge from the hospital continued a 12 weeks home ET program (read in Methods section “Inpatient rehabilitation and home-based program protocols”). IG participants received telephone calls every other week and were asked to answer questions regarding their health and physical activity. Adverse events (AEs) were monitored throughout the exercise intervention in accordance with CONSORT guidelines. AEs were defined as any unfavorable sign, symptom, or injury occurring during or after exercise. Active surveillance was conducted at each session by trained staff, with additional spontaneous reporting during patient time in hospital, and through telephone calls during IG follow-up time.

**Table 1:** Patients inclusion and exclusion criteria

Inclusion criteria	Exclusion criteria
1. Patients admitted to CR after open heart surgery 2. Patient’s agreement to participate in the study 3. Age ≥65 years 4. 6-MWD ≥ 150 m 5. EFS score ≥ 4 points	1. Cardiac devices (due to artificially altered heart rate series) 2. Diseases in the musculoskeletal system or other exercise-limiting comorbidities such as neurological conditions, chronic heart failure (New York Heart Association Class IV), hemoglobin less than 9 g/dL, wound healing disturbance, cognitive or linguistic deficits

### Clinical assessment and outcome measures

Assessment times were: before randomization (admittance to CR) (V1); at CR-completion (V2) with a mean duration of 16.2 ± 2.9 days; and at three months after CR completion (V3) with a mean duration of 104.2 ± 23.0 days. All assessments were blinded and performed by certified staff members who were not involved in clinical care (two medical doctors with a specialization in cardiology, one physiotherapist, and one nurse). The assessment included medical history (i.e. cardiac diagnosis, cardiovascular risk factors, concomitant diseases, as well as at T2 clinical course, events, and hospitalization since the last examination), a clinical examination including veloergometry and measurements of anthropometric data were performed. All assessments included:

1. 6MWT, meters. The test was performed according to the American Thoracic Society [13] to evaluate physical capacity and screen for patient eligibility to participate in the study.
2. Instrumental physical capacity testing, peak workload (W), max. HR beats/minute, MET. The veloergometry was performed on a cycle ergometer using a ramp protocol starting with 25 W and increasing to 12.5 W per minute until subjective exhaustion or occurrence of abort criteria. MET values were calculated based on the results obtained from veloergometry using standard estimation equations.
3. TUG, seconds. The test was used to assess a patient’s functional capacity and mobility. During the test, the time in seconds is measured that is required for the patient to rise from a chair, walk 3 m away back and forth, and sit down again.
4. Frailty was evaluated with EFS score [17], and frailty status. EFS involves nine domains of frailty including cognition, general health status, functional independence, social support, medication usage, nutrition, mood, continence, and functional performance. During this study, the patients were classified into the following categories as suggested on the official EFS website (Edmontonfrailscale.org): fit (score of 0 to 3), vulnerable (score of 4 to 5), and frail (score of 6 to 17).

### Inpatient rehabilitation and home-based program protocols

An individual rehabilitation program was created for each patient, which was compiled by a doctor of physical medicine and rehabilitation. All patients participated in a physical therapy program, which included inspiratory muscle training (IMT) exercises (7 days/week 15 min duration), aerobic constant workload training (6 days/week, 30 min, 30–60% watt max, 60–70% maximal heart rate), and relaxation exercises (6 days/week 15 min). During CR IG patients received additional sensomotoric and muscle strength training sessions to familiarize themselves with the home-based program which intended to last for 12 weeks. While CG patients were asked just to maintain physical activity, IG patients received a detailed ET program for home-based rehabilitation.

Exercise training at home included (1) aerobic endurance training (moderate-intensity aerobic activity for a minimum of 30 min per day 5 times a week or high-intensity for a minimum of 20 min 3 times a week). Proper activities for aerobic training at home were climbing stairs, walking, cycling (2) sensomotoric training (includes postural control, dynamic balance, coordination, flexibility), (3) muscle strength training (moderate-intensity activities are recommended to maintain or increase muscle strength), and (4) flexibility exercises (low to moderate isolated type exercises or integrated exercises that include neck, legs, arms stretching exercises). It was recommended to perform 8–10 exercises (10–20 repetitions) (involving the main muscle groups of the legs and arms). In physical therapy sessions weights (starting from 0.5 to 2.0 kg), elastic resistance bands (The color of the resistance bands indicates the level of resistance), and unstable balance platforms (with different foam surfaces) were used. A structured control and intervention home-based program is provided in the Supplement material (Table A6, A7).

IG participants received telephone calls every other week and were asked to answer questions regarding their general and cardiovascular health, general physical activity, exercise training program implementation or obstacles to continue training. Exercise intensity fidelity was assessed based on participants’ self-reported training heart rate and perceived exertion using the Borg scale. Participants were able to consult directly with a physiotherapist or medical doctor if needed.

### Statistical analysis

Statistical analyses were performed using SPSS version 22 to evaluate the effects of the intervention on functional and clinical outcomes. Normality was assessed using the Shapiro–Wilk and Kolmogorov–Smirnov tests. Non-parametric tests were primarily used due to non-normal distributions: the Mann–Whitney *U* test for unpaired between-group comparisons of change scores (Δ V2–V1), the chi-square test for categorical baseline variables such as gender, and the Friedman test for within-group repeated-measures changes. Unpaired Student’s *t*-tests were used where appropriate.

Frailty stage analyses were conducted using a complete case approach, including only participants with available data at both baseline and follow-up assessments. Missing follow-up data were not imputed. Given the pilot nature of the study and the relatively small sample size, this approach was chosen to avoid introducing additional assumptions regarding missing data.

Baseline imbalances in gender distribution were observed; however, formal adjustment for baseline values and gender was not included in the primary analyses due to the exploratory nature of the study. As a sensitivity analysis, post hoc ANCOVA adjusting for baseline values and gender was performed for selected key outcomes (e.g., the 6-minute walk test) and confirmed the observed trends. All statistical tests were two-sided, and a *p* value < 0.05 was considered statistically significant.

## Results

Out of 336 patients assessed for eligibility on the first day of admittance to the rehabilitation hospital. 236 were excluded from the sample because they did not conform to the inclusion criteria (their 6MWT was lower than 150 m, age less than 65 years, EFS less than 4, some had comorbidities limiting exercising, cognitive or mental disorders, implanted pacemaker or non-healing wounds and 9 patients refused to participate in the study). 100 (38 females, 62 males) patients 17.92 ± 11.94 days post-surgery) fulfilled the inclusion criteria (Table 1). and were randomly assigned to the IG and CG. The groups were generally well-matched except there were considerably more men in the control group (Table 2).

**Table 2:** Demographic and clinical characteristics in the intervention and control groups before inpatient cardiac rehabilitation

	Intervention Group	Control Group	*P* value
Female	25 (50%)	13 (26%)	0.013*
Male	25 (50%)	37 (74%)	
Age, years	73.2 ± 4.8	73.4 ± 5.3	0.831
Height, cm	165.9 ± 8.6	169.4 ± 8.6	0.046
Weight, kg	74.9 ± 12.8	78.7 ± 13.2	0.151
Body mass index, kg/m2	27.3 ± 4.8	27.4 ± 3.9	0.93
Post – surgery, days	16.6 ± 7.3	17.6 ± 7.5	0.469
Surgery			
CABG	23 (46%)	33 (66%)	
Isolated valve	11 (22%)	5 (10%)	
Combined	16 (32%)	12 (24%)	
Heart Failure Class			0.391
NYHA I	2 (4%)	3 (6%)	
NYHA II	40 (80%)	34 (68%)	
NYHA III	8 (16%)	13 (26%)	
Comorbidities			
Atrial fibrillation			0.213
Paroxysmal	7 (14%)	14 (28%)	
Persistent	2 (4%)	1 (2%)	
Chronic	6 (12%)	2 (4%)	
COPD	0	3 (6%)	0.079
Depression	1 (2%)	2 (4%)	0.617
Musculoskeletal system disease	1 (2%)	3 (6%)	0.362
Oncological disease	4 (8%)	8 (16%)	0.357
Diabetes	7 (14%)	10 (20%)	0.242
Hypertension	44 (88%)	48 (96%)	0.269
Functional capacity			
Peak workout, W	48.49 ± 15.8	52.2 ± 17.6	0.278
6 min walk test, m	289 ± 86.1	291 ± 79.6	0.861
Timed up ang go test, s	8.9 ± 2.4	8.5 ± 1.7	0.301
Edmonton frail scale score	6.18 ± 1.6	5.98 ± 1.6	0.558
Distribution of frailty categories			0.944
Robust, %	0	0	
Prefrail, %	20 (40%)	21 (42%)	
Frail, %	30 (60%)	29 (58%)	

*gender distribution differences between the groups (CG vs. IG)

Both groups participated in exercise training during 20 days inpatient-CR. 91 patients (IG *n* = 45, CG *n* = 46) finished the study during their stay in inpatient-CR (9 patients dropped out because 7 patients refused further participation, and 2 patients had been infected of Covid-19). No AE was registered during the CR phase. Elderly pre-frail and frail patients after cardiac surgery tolerated the tailored exercise program well, relapse was rare, and all 7 patients who withdrew from the study did it at the very beginning. No AE related to exercise training were observed. The IG performed an average of 37.27 ± 3.58 sessions overall, while the CG performed 33.40 ± 6.19 sessions (*p* = 0.0005), as IG patients had additional sessions for preparation to participate in the home training program.

As a result of inpatient-CR all measured parameters except max. HR enhanced significantly in both groups. Higher 6MWT and TUG results in both groups (*p* < 0.001) which indicated increased functional capacity and improved mobility. EFS scores decreased in both groups (*p* < 0.001) indicating reduced frailty. Maximal workload improved in both groups: by 8.59 ± 8.54 W in CG and 11.49 ± 9.51 W in IG (*p* = 0.001 and *p* < 0.001). MET increased in both groups with a significant within-group change for IG (*p* = 0.001). Analyzing between-group differences at the second evaluation, the intervention group demonstrated numerically greater improvements across multiple outcomes compared with the control group, including 6MWT (+ 12.4 m vs. + 8.2 m, *p* = 0.205), TUG (− 0.8 s vs. − 0.4 s, *p* = 0.114), and EFS (− 1.6 vs. − 0.8, *p* = 0.082). A statistically significant between-group difference was observed only for physical capacity measured by MET (*p* = 0.037), with a mean difference of − 47.5 (95% CI − 93.4 to − 1.6). Detailed clinical outcomes are presented in Table 3.

**Table 3:** Clinical outcomes at three time points (V1: baseline, V2: post-rehabilitation, V3: follow-up) in Control Group (CG) and Intervention Group (IG)

	V1			V2			V3		
	CG	IG	*P* value	CG	IG	*P* value	CG	IG	*P* value
6 MWT	291.42 ± 79.56	288.50 ±86.04	0.861	363.76 ± 91.18	389.39 ± 99.93	0.205	426.42 ± 97.59	451.29 ± 83.44	0.304
EFS	6.14 ± 1.91	6.13 ± 1.62	0.558	5.08 ± 2.03	4.47 ± 1.83	0.082	4.52 ± 1.69	4.06 ± 1.81	0.335
TUG	8.50 ± 1.69	8.93 ± 2.38	0.563	7.93 ± 1.80	7.47 ± 1.76	0.114	8.46 ± 2.07	8.27 ± 3.19	0.361
Max Watt	52.16 ± 17.62	48.49 ± 15.79	0.595	60.37 ± 21.76	64.00 ± 20.11	0.230	77.57 ± 30.16	72.79 ± 17.04	0.988
Max MET	3.07 ± 0.77	3.10 ± 0.89	0.605	3.40 ± 1.01	3.84 ± 1.12	**0.037**	4.04 ± 1.32	4.27 ± 1.04	0.141
Max HR	97.52 ± 21.00	91.19 ± 12.87	0.795	96.41 ± 16.34	93.57 ± 15.74	0.638	108.43 ± 19.00	100.21 ± 12.54	0.742

Values are presented as mean ± standard deviation

*6MWT* 6-Minute Walk Test, *EFS* Edmonton Frailty Scale, *TUG* Timed Up and Go, *MET* Metabolic Equivalent, *HR* Heart Rate, *CG* Control Group, *IG* Intervention Group

*P*-values indicate between-group differences at each time point

58 patients attended and completed the entire study (IG *n* = 33, CG *n* = 25) and were evaluated all three times, while 33 patients dropped out from the trial. The reasons for not participating till the end of the study were mainly lack of social support (29 patients), health deterioration (2 patients) and 2 patients were lost to follow-up (Fig. 1). IG patients were able to successfully participate in the home training program and followed physical activity recommendations. None of the patients discontinued participation in the telephone call program or reported health deterioration connected to additional exercise, no AE were registered. To assess potential bias related to patient drop-out, we compared baseline characteristics between participants who returned for follow-up (*n* = 58) and those who did not (*n* = 33). CG and IG number of drop out patients was not significantly different (*p* = 0.211). An independent sample t-test showed no statistically significant difference in EFS scores between the groups (*p* = 0.76), age (*p* = 0.916) and gender (Table A8).

The 6MWT results after 12 weeks increased in both groups, but not significantly (*p* = 0.322 IG; *p* = 0.057 CG), and no significant difference between the groups was observed (*p* = 0.304). Physical capacity measurements (max. Load, MET) improved significantly in both groups (both *p* < 0.001) with no significant differences in between. Regardless of the TUG result decreased during the last (from V2 to V3) period in both groups (from 7.47 ± 1.76 to 8.27 ± 3.19 s in the IG and from 7.93 ± 1.80 to 8.46 ± 2.07 s in the CG), EFS score improved for all patients over time, without significant differences among groups. IG patients showed numerically higher but not statically significant results compared to CG in 6MWT, EFS score, TUG, and MET at the third assessment.

One of the study aims was to assess the feasibility and preliminary performance of the Edmonton Frail Scale (EFS) for detecting changes in frailty status over a 3-month follow-up period. According to the EFS score 60% in CG and 58% in IG patients were frail at the baseline (*p* = 0,955). Frailty status improved in both groups at V2 (*p* < 0.001), without statistical significance in-between and sustained at V3 (*p* = 0,398). (Table 4). Among complete case participants (CG *n* = 25, IG *n* = 33), the majority were frail at baseline (CG 72%, IG 73%). In the complete case analysis (Table A9), frailty decreased significantly within both groups from V1 to V3 (CG: 75%→32%, *p* < 0.01; IG: 73%→12%, *p* < 0.001; McNemar test), with greater improvement in IG. At V3, IG showed higher robust (21% vs. 14%) and prefrail (67% vs. 54%) proportions despite similar baseline frailty. No significant between-group differences were observed (χ² *p* = 0.32).

**Table 4:** Distribution of frailty categories based on Edmonton Frailty Scale (EFS) scores across three time points (V1, V2, V3) in Control Group (CG) and Intervention Group (IG)

EFS scoring	EFS score frequency V1		EFS score frequency V2			EFS score frequency V3	
	CG	IG	CG	IG	CG		IG
Robust (1–3)	0	0	9 (18%)	14 (28%)	9 (18%)		15 (30%)
Prefrail/vulnerable (4–5)	20(40%)	21 (42%)	19 (38%)	22 (44%)	9 (18%)		12 (24%)
Frailty (6–17)	30 (60%)	29 (58%)	17 (34%)	10 (20%)	7 (14%)		6 (12%)
Missing patient	0	0	5 (10%)	4 (8%)	25 (50%)		17 (34%)
*P* value between groups	0.994		0.062			0.257	
	CG V1/V2	IG V1/V2	CG V1/V3	IG V1/V3		IG V2/V3	CG V2/V3
*P* value in time	**0.02**	**0**	**0**	**0**		0.389	0.388
*P* value in time between groups	0.955		0.606			0.136	

*P***-**values show between-group comparisons at each time point and changes over time. Categories: Robust (1–3), Pre-frail (4–5), Frail (6–17)

**Fig. 2:**
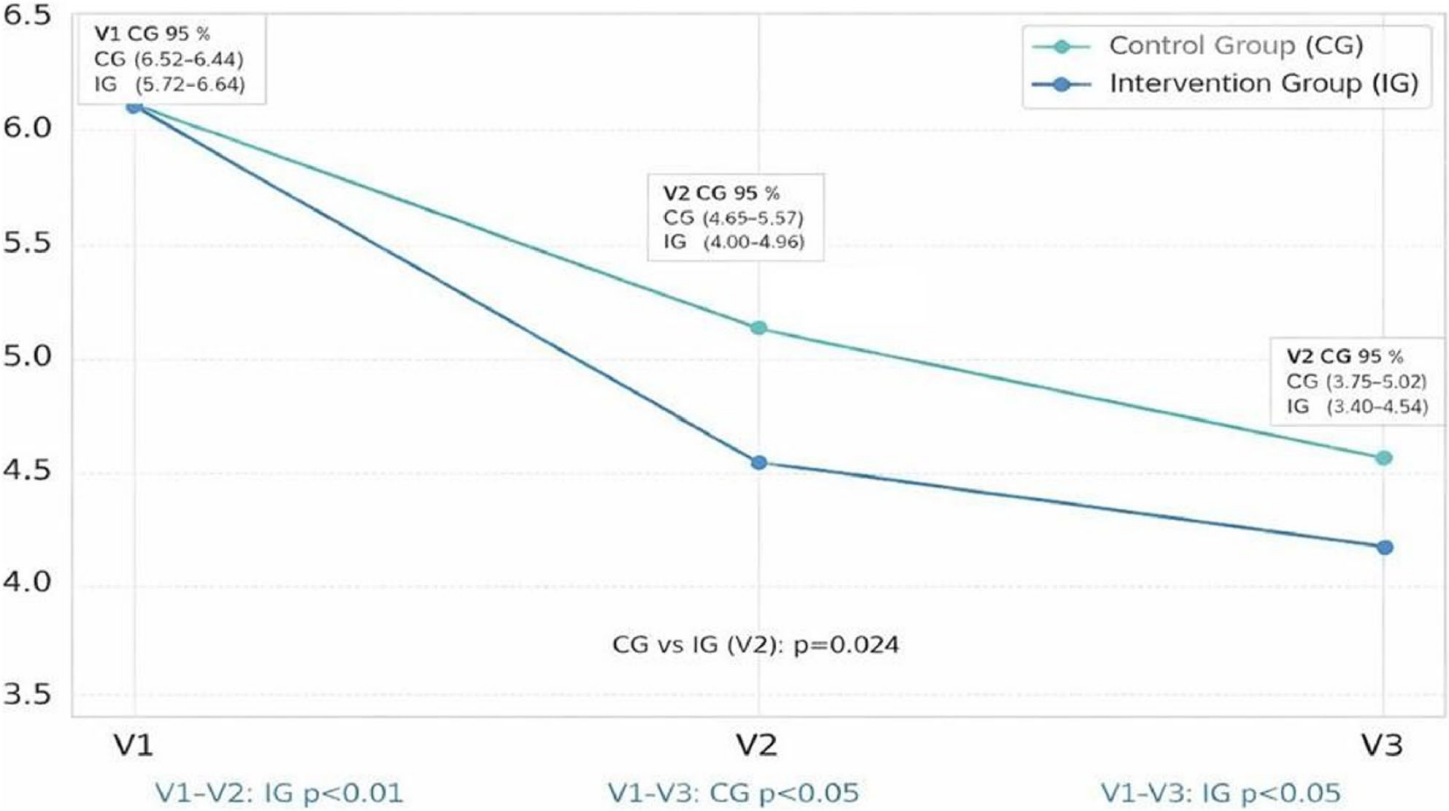
Mean Edomonton Frail Scale score Over Time

**Fig. 3:**
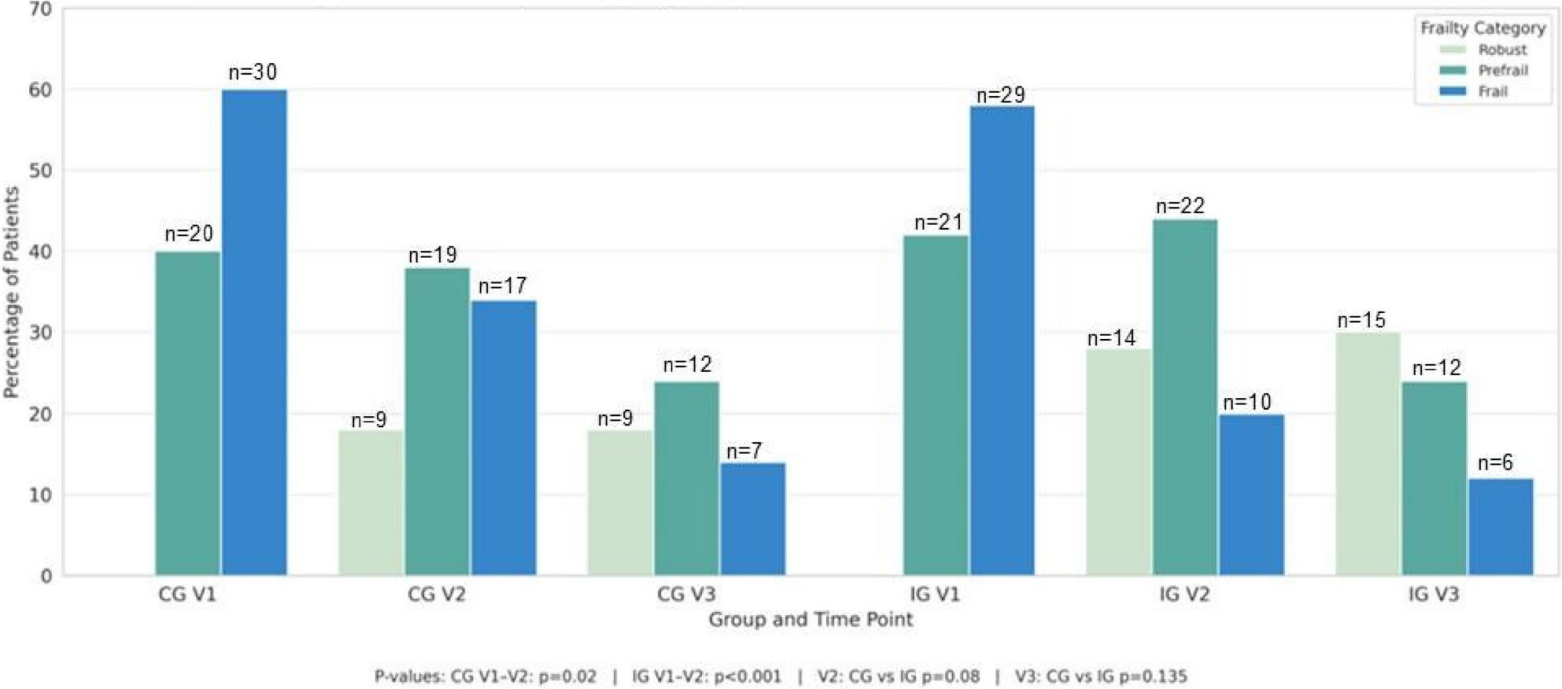
Frailty category distribution over time in CG and IG

A detailed analysis of individual frailty domains shows alterations in specific aspects between the groups across the visits (Table 5). Only the general health domain was statistically significantly higher at follow-up visit in compared to CG (*p* = 0.03), also general health status improved significantly in both groups over time, with more pronounced changes in the IG (*p* < 0.001 for both V1–V2 and V1–V3), and significant between-group differences (*p* < 0.001). Statistically significant improvements over time were observed in several frailty domains both within the IG and CG in time. Cognitive function improved significantly during the time only in the IG from V1 to V2 (*p* = 0.024), also there was greater cognitive function compared over time in IG at both V1–V2 (*p* = 0.009) and V1–V3 (*p* = 0.027). Moreover, improvements in mood and functional performance were observed in the IG, with significant changes between V1–V2 (both *p* < 0.001) and V1-V3 (*p* = 0.01 and *p* = 0.005 respectively) besides a significant differences between, favoring the IG were confirmed (V1-V2 mood *p* = 0.008, functional performance *p* < 0.01; V1-V3 mood *p* = 0.013, functional performance *p* < 0.01)). Domains such as social support and continence showed no significant changes over time or between groups. No significant differences were observed in V2-V3 in all frailty domains for both groups. Changes in EFS over time in IG and CG are represented in Fig. 2 while a comparison of EFS trends between groups is visible in Fig. 3.

**Table 5:** Mean scores of EFS subdomains over time (V1, V2, V3) for Control Group (CG) and Intervention Group (IG)

	V1			V2			V3			*P* value in time between groups			
	CG	IG	*P* value	CG	IG	*P* value	CG	IG	*P* value	CG vs. IG V1-V2	CG vs. IG V1-V3		CG vs. IG V2-V3
Cognition	1.54 ± 0.791	*1.62 ± 0.68	0.643	1.15 ± 0.93	*1.3 ± 0.89	0.462	1.19 ± 0.83	1.22 ± 0.91	0.884	**0.009**	**0.027**	0.556	
General health status	*2 ± 0.61	*1.83 ± 0.70	0.221	*1.59 ± 0.6	*1.35 ± 0/53	0.056	**1.63 ± 0.84	**1.25 ± 0.44	**0.030**	**< 0.001**	**< 0.001**	0.346	
Functional independence	0.41 ± 0.62	*0.38 ± 0.53	0.830	0.38 ± 0.54	*0.26 ± 0.44	0.241	0.33 ± 0.48	0.31 ± 0.54	0.877	0.058	0.568	0.819	
Social support	0.05 ± 0.21	0.02 ± 0.15	0.524	0.05 ± 0.22	0	0.136	0	0.03 ± 0.18	0.363	1	1	1	
Medication use	0.95 ± 0.61	0.96 ± 0.72	0.984	1.03 ± 0.54	0.95 ± 0.69	0.601	**0.81 ± 0.56	0.84 ± 0.52	0.837	0.225	0.072	0.464	
Nutrition	0.41 ± 0.5	0.23 ± 0.43	0.075	0.36 ± 0.49	0.3 ± 0.47	0.591	0.07 ± 0.27	0.09 ± 0.3	0.791	0.774	**0.013**	**0.007**	
Mood	0.2 ± 0.41	*0.4 ± 0.5	**0.040**	0.28 ± 0.46	*0.14 ± 0.35	0.115	0.11 ± 0.32	**0.19 ± 0.4	**0.425**	**0.008**	0.035	1	
Continence	0.11 ± 0.32	0.09 ± 0.28	0.653	0.1 ± 0.31	0.09 ± 0.29	0.886	0.19 ± 0.4	0.03 ± 0.18	0.052	1	0.727	0.289	
Functional performance	*0.45 ± 0.55	*0.53 ± 0.58	0.517	*0.13 ± 0.34	*0.07 ± 0.26	0.379	**0.19 ± 0.4	**0.09 ± 0.39	**0.377**	**< 0.01**	< 0.01	0.414	

*CG* Control Group, *IG* Intervention Group, *EFS* Edmonton Frailty Scale

*p*-values refer to between-group comparisons at each time point and changes over time

*p < 0.05 for V1–V2 comparison, **p < 0.05 for V1–V3

## Discussion

This pilot branch study aimed to assess the feasibility and preliminary performance of the Edmonton Frail Scale (EFS) for detecting changes in frailty status over a 3-month follow-up period after cardiac surgery in older adults, and to explore frailty within the context of exercise-training–based cardiac rehabilitation. 

Our pilot study results demonstrated that EFS is a suitable tool and may be used for tracking frailty for elderly patients in a context of CR early after heart surgery. There was significant drop-out at V3 assessment and study failed to demonstrate reliable and significant changes in functional and physical capacity. On the other hand, when IG participants could not participate due to lack of social support or COVID lockdown, we were able to reach them through telephone calls to register AEs, training intensity and answer questions regarding general and cardiovascular health. Additional exercise training and extended follow-up, during the home-based rehabilitation program was feasible and safe in this vulnerable patient population.

All study participants showed statistically significant reductions in total EFS scores following inpatient CR at V2 and after 3 months follow-up at V3. Also, greater reductions in frailty scores, particularly in the domains of general health status and functional performance were detected in IG patients. Even modest improvements in EFS can reflect meaningful gains in physical function, reduced fall risk, improved mood or greater independence, which is highly relevant in older adults undergoing cardiac rehabilitation [18]. These findings are consistent with recent studies indicating that structured, multi-component exercise interventions can reduce frailty and improve clinical outcomes in post-surgical cardiac patients [10, 19, 20]. Noteworthy, the 2024 ESC guidelines for the management of chronic coronary syndrome and the 2023 ESC guidelines for the management of acute coronary syndromes acknowledge frailty as a key factor influencing primary syndromes and outcomes in cardiac [21, 22]. Frailty is now recognized as one of the critical variables in such decision-making, as these patients often face elevated mid-term mortality risk although specific guidance for managing frailty in the context of cardiac surgery remains underdeveloped. Our pilot study contributes valuable insights into this area, suggesting that patients that were frail or pre-frail early after cardiac surgery can change their frailty status in 20 days and 3 month follow up periods and EFS can be used to monitor that change.

While rehabilitation models may vary slightly across countries, most studies implement interventions lasting between 8 and 12 weeks, particularly when targeting older populations [23, 24]. The 12-week duration of the home-based exercise program in our study aligns with common clinical practice and has been demonstrated in several studies to be sufficient for participants to develop and maintain exercise habits independently [25–27]. Such a time frame allows for physiological improvements and behavioral adaptations necessary for sustained physical activity. In our study 12 - week ET program consisted of aerobic and resistance training combined with balance, flexibility, sensomotoric and respiratory exercises as evidence-based approaches for reversing or preventing frailty. The IG patients, who received an extended 12-week home-based training program, showed higher but not statistically significant results compared to CG in 6MWT, EFS score, TUG and MET at the third assessment. Involvement of various training, with functionally diverse effects across domains of the EFS (for example mood, general health and functional performance) may be led to statistically significant general improvement, greater in the intervention group. Furthermore, the study design included objective functional assessments (6MWT, TUG, MaxWatt, MET, HRmax), which we believe offered strong physiologic metrics to validate subjective findings related to frailty scores.

At the third evaluation point, a slight regression in mobility scores (TUG test in seconds) was observed, particularly in the CG. Several hypotheses may explain this finding. First, the discontinuation of structured exercise following inpatient rehabilitation may have led to deconditioning. Previous studies have shown that patients who do not continue regular physical activity after structured rehabilitation experience a decline in functional capacity within a few weeks [28–30]. Second, reduced adherence to physical activity guidelines, especially in the absence of supervision, may have contributed to the loss of previously achieved improvements. By contrast, the intervention group, which maintained a prescribed exercise routine with periodic follow-up, demonstrated greater stability. This is in line with evidence showing that long-term adherence to physical activity significantly reduces the recurrence of frailty and helps sustain functional gains over time [7]. On the other hand we were not able to evaluate long term outcomes such as hospitalization and/or mortality. These findings underscore the need for larger-scale studies to further validate and expand upon these results [19, 22].

According to other authors, it remains unclear whether engagement in regular physical activity of sufficient intensity could, at least partially, substitute for planned exercise training in elderly patients following cardiac surgery. Recent evidence suggests that home-based or hybrid cardiac rehabilitation models and increases in habitual physical activity can lead to improvements in physical function, frailty reduction and selected patient-reported outcomes in older cardiac populations, particularly among individuals unable or unwilling to participate in center-based programs [31–34]. However, randomized trials and systematic reviews have clearly demonstrated that structured exercise training continues to be superior in increasing cardio-respiratory fitness and exercise capacity [35], whereas benefits from general physical activity seem to be less impressive and varying [36]. Importantly, studies in very old and frail patients have shown that improvements in functional performance and frailty status may occur over time even in the absence of robust between-group differences in fitness outcomes, highlighting the potential contribution of overall activity levels [37]. Taken together, these findings suggest that while regular physical activity may represent a pragmatic and valuable component of post-operative recovery—particularly from a feasibility and adherence perspective—it cannot currently be considered an equivalent replacement for structured exercise training.

There are several limitations that need to be considered. The most important - were a relatively small study sample and the high dropout rate (33%). The study is characterized by inadequate power for conducting between-group comparisons at the follow-up stage. It is possible that non-significant findings may be indicative of a false negative type II error. This study is a pilot trial intended to generate preliminary data and inform the design of future, larger-scale investigations with more robust statistical power. The sizes of the groups and inclusion period in this particular project were defined according to project timeline and funding. Additionally, the study was conducted at a single centre, which—while allowing for uniform implementation of the intervention and follow-up regimes, it might limit the generalizability of the results to other settings or healthcare systems. Multicentre studies will be necessary to confirm the broader applicability of these results. The high dropout rate in our study can be related to several interconnected factors as characteristics of the study population and external circumstances during the observation period. Importantly, patient monitoring coincided with the COVID19 pandemic, during which government restrictions, lockdowns and fear of infection among older adults further limited participants mobility. Our target group consisted of older adults who often required assistance from others and a significant proportion of these individuals lived far from the hospital where the assessments were conducted. Basically, these patients were unable to travel independently and had logistical challenges such as lack of transportation, these reasons may have led to reduced motivation to attend to follow up assessments. While visits were scheduled they could have been considered as not essential by patients, particularly in cases where no major changes in health status were noted. It is also worth highlighting that IG patients received biweekly phone calls to assess their progress and encourage adherence to the exercise program. We strongly believe that this consistent communication may have contributed to the relatively higher retention rate in the IG compared to CG. Based on these observations future studies could benefit from incorporating remote or hybrid (in-person, telehealth) monitoring strategies. Regular virtual consultations, supported by phone and home-based assessment could significantly enhance adherence, reduce drop rates and improve feasibility of similar studies, particularly when dealing with older and limited mobility populations.

Another important limitation was gender imbalance among groups. However, current evidence suggests that sex does not independently influence recovery, adherence or outcomes in frail cardiac patients. Louise Y Sun et al. (2022) [38], demonstrated that age, not sex modifies the effect of frailty on long term outcomes after cardiac surgery. Similarly, a 2022 meta-analysis found no significant gender - related differences in functional improvements following cardiac rehabilitation. Therefore, while noted, this demographic difference is unlikely to have confounded our primary results.

This pilot study suggests that structured, individualized, and partially home-based CR may represent a feasible and safe approach with potential to positively influence frailty and functional status in elderly patients after cardiac surgery. However, these findings should be interpreted with caution, and further research in larger populations and diverse healthcare settings is needed to confirm and expand upon these preliminary observations.

## Conclusions

This study explored the responsiveness of the Edmonton Frail Scale (EFS) to short-term changes in frailty status among elderly patients recovering from cardiac surgery. Within-group analyses indicated that EFS scores changed over time, suggesting that the instrument is capable of capturing short-term variation in frailty status in this clinical context. However, in the absence of comparison with an external reference or gold standard measure of frailty, conclusions regarding the validity of these changes should be interpreted with caution. The structured cardiac rehabilitation program, including a home-based exercise component, was found to be feasible and safe in this high-risk population. Although reductions in frailty scores were observed over time within groups, between-group differences were not consistently demonstrated, and the study was not powered to establish intervention effectiveness. The high dropout rate further limited the ability to draw definitive conclusions regarding the impact of the rehabilitation program on frailty or physical capacity outcomes. Future adequately powered, multi-center studies incorporating comparator instruments or anchor-based approaches are needed to confirm the responsiveness of the EFS and to clarify the effects of structured rehabilitation on frailty trajectories in this population.

## Data Availability

The subset of the database used in the study will be made available at PhysioNet.

## Appendix A

**Table 6:** Home-based program for Intervention group (12 Weeks program)

Training time and type	Frequency	Intensity	Time	Exercises
**First month**				
**Aerobic activity**	6 days/week	moderate-high, Borg 12-14	30-40 min.	Proper activities are climbing stairs, walking, cycling.
**Sensomotoric training**	3 days/week.	moderate-high, Borg 11-12	15 min.	Includes postural control, dynamic balance, coordination, flexibility. All exercises 3 sets 10 repetitions. 1) While sitting, place your feet on a cushion, twisted towel, or unstable surface (if you have one). Perform the exercise with your eyes closed. 2) Stand up. Keep your legs wide, shoulder-width apart. Close your eyes. Hold 5 sec. 3) Stand up. Keep your legs wide, shoulder-width apart. Close your eyes. Place one leg forward in relation to the other leg. Stand for 5 seconds and do the same with the other leg. 4) Stand up. If you can, do the exercise without sticking, if you can't, hold on to a chair or other stable surface. Keep your legs wide, shoulder-width apart. Place one foot on an unstable surface pad, twisted towel, or unstable surface (if you have one). Close your eyes. Transfer the weight from one leg to the other. 5) When standing up, raise your right leg (bend your leg back) and hold it for 30 seconds, do the same with the other leg.. 6) When standing, raise your right leg at a right angle, hold it for 30 seconds, do the same with the other leg. 7) When standing up, walk one straight line foot by foot.
**Muscle strength**	3 days/week.	<30% 1MR (maximum repetition)	20-25 min.	It is recommended to perform all exercises 3 sets 10 repetitions. involving the main muscle groups of the legs and arms. 1) When standing up, lift your heels completely off the floor. Exhale during exercise (ascending), inhale after descending. Do the exercise without holding. 2) While sitting on the chair, lift your heels. When stretching one leg at a time, give yourself resistance with your hand at the knee or use weights on the ankles (0.5 kg). 3) Sit on the chair. Take the ends of the resistance tape in your hands and fold the rest of the tape over the middle of the sole. With exhalation, stretch your leg forward. Do the same exercise only without bending the knee, and the resistance is given only to the toes (the calf muscles work). Use the red resistance bar. 4) Sit on the chair. Place the ball between your knees and try to exhale by pressing the ball or a compressed towel between your knees. Do not hold your breath. 5) Sit on the chair. Place the ball between your inner ankles. Keeping the distance between your knees with the inside of your feet and exhaling, try to compress the ball. 6) Sit on the chair. Wrap your thighs in a resistance band. With the feet compressed, perform a hip abduction. Use the red resistance bar. 7) While sitting, lift one leg up. Give yourself resistance at the knee with your hand or use weights on the ankles (0.5 kg). 8) Sit in a chair. Take the ball in your hands and press gently (do not hold your breath).
**Flexibility**		low-moderate, Borg 11-12	10 min.	Low to moderate isolated type flexibility exercises or integrated flexibility exercises. Includes leg, arm, neck stretching exercises. All exercises 3 sets 10 repetitions. 1) Stand against the wall, one foot in front, the other foot in the back. The hind leg must always remain straight. When bending the front leg with exhalation along the front leg path, try to reach the wall. Do not stretch, the body must remain in line. When the exercise is done correctly, there is a feeling of tension in the calf of the hind leg. Hold 5 sec. 2) Sit on the chair. One leg bent, the other leg straight. The most important thing is that the knees of the streching leg are not bent, if stretching is tolerated, the toes can be tilted towards you. Hold 5 sec.
**Second month**				
**Aerobic activity**	6 days/week	moderate-high, Borg 12-14	30-40 min.	Proper activities are climbing stairs, walking, cycling.
**Sensomotoric training**	3 days/week.	moderate-high, Borg 11-12	15 min.	Includes postural control, dynamic balance, coordination, flexibility. All exercises 3 sets 10 repetitions. 1) While sitting, place your feet on a twisted towel, or unstable surface (if you have one). Perform the exercise with your eyes closed and place acushion under your gluteus (or if you have an unstable plate). 2) Stand up. Keep your legs wide, shoulder-width apart. Close your eyes. Try typing a number from 1 to 10 with your finger. 3) Stand on an unstable plate or surface. Keep your legs wide, shoulder-width apart. Close your eyes. Place one leg forward in relation to the other leg. Stand for 5 seconds and do the same with the other leg. 4) Stand up. Hold the chair or other stable surface. Climb on an unstable surface pad, a twisted towel, or an unstable surface (if you have one). Stand for 30 seconds. Close your eyes. 5) When standing on an unstable plate or pillow, lift your right leg (bend your leg back) and hold it for 30 seconds, do the same with the other leg. 6) When standing up, go one straight line foot behind the foot. 7) When standing up, walk one straight line with your foot behind your foot on an unstable surface (on pads).
**Muscle strength**	3 days/week.	30-50% 1MR (repetition maximum)	20-25 min.	It is recommended to perform all exercises 3 sets 10 repetitions. involving the main muscle groups of the legs and arms. 1) Lift your heels completely off the floor. Exhale during exercise (ascending), inhale after descending. Do the exercise without adhering. You can put on a weight of 1.0 kg. 2) While sitting on the chair, make uprights. Give yourself resistance with your hand at the knee or use weights on the ankles (1.0 kg). 3) Sit on the chair. Take the ends of the resistance tape in your hands and fold the rest of the\ tape over the middle of the sole. With exhalation, stretch your leg forward. Do the same exercise only without bending the knee, and the resistance is given only to the toes (the calf muscles work). Use the green resistance band. 4) Sit on the chair. Place the ball between your knees and try to exhale by pressing the ball or a compressed towel between your knees. Do not hold your breath. 5) Sit on the chair. Place the ball between your inner ankles. Keeping the distance between your knees with the inside of your feet and exhaling, try to compress the ball. 6) Sit on the chair. Wrap yourthighs in a resistance band. With the feet compressed, perform a hip abduction. Use the green resistance band. 7) When standing up, lift one leg up. Hold on if you can use the green resistance bar. 8) Sit on the chair. Take the ball in your hands and press gently (do not hold your breath).
**Flexibility**		low-moderate, Borg 11-12	10 min.	Low to moderate isolated type flexibility exercises or integrated flexibility exercises. Includes leg, arm, neck stretching exercises. 1) Stand against the wall, one foot in front, the other foot in the back. The hind leg must always remain straight. When bending the front leg with exhalation along the front leg path, try to reach the wall. Do not stretch, the body must remain in line. When the exercise is done correctly, there is a feeling of tension in the calf of the hind leg. Hold 5 sec. 2) Sit on the chair. One leg bent, the other leg straight. The most important thing is that the knee of the extended leg is not bent, if stretching is tolerated, the toes can be tilted towards you. Hold 5 sec.
**Third month**				
**Aerobic activity**	6 days/week	moderate-high, Borg 12-15	30-40 min.	Proper activities are climbing stairs, walking, cycling.
**Sensomotoric training**	3 days/week.	moderate-high, Borg 11-12	15 min.	Includes postural control, dynamic balance, coordination, flexibility. All exercises 3 sets 10 repetitions. 1) When standing, place your feet on a pillow, twisted towel, or unstable surface (if you have one). Try to stand, if it is not hard, close your eyes. 2) Stand up. Keep your legs wide, shoulder-width apart. Close your eyes. Try typing a number from 1 to 10 with your finger. 3) Stand on an unstable plane or pillow. Keep your legs wide, shoulder-width apart. Close your eyes. Place one leg forward in relation to the other leg. Stand for 5 seconds and do the same with the other leg. 4) Stand up. Try to do the exercise without adhering. Climb on an unstable surface pad, twisted towel, or unstable surface (if you have one). Stand for 30 seconds. Close your eyes. 5) When standing up, go one straight line foot behind the foot. 6) When standing up, walk one straight line with your foot behind your foot on an unstable surface (on pads).
**Muscle strength**	3 days/week.	moderate (30-60% 1RM), Borg 12-15	20-25 min.	It is recommended to perform all exercises 3 sets 10 repetitions. involving the main muscle groups of the legs and arms. 1) When standing up, lift your heels completely off the floor. Exhale during exercise (ascending), inhale after descending. Do the exercise without adhering. You can put on a weight of 1.0 kg. If it’s not hard, don’t lower your heels all the way down. 2) While sitting on the chair, raise your heels. When stretching one leg at a time, give yourself resistance with your hand at the knee or use weights on your ankles (2.0 kg). 3) Sit on the chair. Take the ends of the resistance tape in your hands and fold the rest of the tape over the middle of the sole. With exhalation, stretch your leg forward. Do the same exercise only without bending the knee, and the resistance is given only to the toes (the calf muscles work). Use the blue resistance band. 4) Sit on the chair. Place the ball between your knees and try to exhale by pressing the ball or a compressed towel between your knees. Do not hold your breath. 5) Sit on the chair. Place the ball between your inner ankles. Keeping the distance between your knees with the inside of your feet and exhaling, try to compress the ball. 6) Sit on the chair. Wrap your thighs in a resistance band. With the feet compressed, perform a gluteus abduction. Use the blue resistance bar. 7) When standing up, lift one leg up. Hold on if you can use the blue resistance bar. 8) Sit in a chair. Take the ball in your hands and press gently (do not hold your breath).
**Flexibility**		low-moderate, Borg 11-12	10 min.	Low to moderate isolated type flexibility exercises or integrated flexibility exercises. Includes leg, arm, neck stretching exercises. All exercises 3 sets 10 repetitions. 1) Stand against the wall, one foot in front, the other foot in the back. The hind leg must always remain straight. When bending the front leg with exhalation along the front leg path, try to reach the wall. Do not stretch, the body must remain in line. When the exercise is done correctly, there is a feeling of tension in the calf of the hind leg. Hold 5 sec. 2) Sit on the chair. One leg bent, the other leg straight. The most important thing is that the knee of the extended leg is not bent, if stretching is tolerated, the toes can be tilted towards you. Hold 5 sec.

**Table 7:** Exercise training program for both usual care (control group) and intervention group

Training time	Type	Frequency	Intensity	Time
secondary inpatient cardiac rehabilitation	Aerobic endurance training 6 days /week (30 min)	6 days/week	From low to moderate continous endurance training. 1) warm up (< 50% target intensity, gradually increasing load 1–10 W/min, time 5–10 min); 2) exercise phase (30–50% watt_max_, or 60–70% maximal heart rate (HR_max_)), up to 20–30 min; 3) cool down with gradual reduction of the load, time up to 5 min.	30–40 min
secondary inpatient cardiac rehabilitation	Aerobic dynamic exercises 5 days/week (30 min); stretching exercises, exercises for the upper and lower limbs	6 days/week	From low to moderate intensity, exercises Borg ≤ 13, 1 min of exercise, 45–60 s rest, from easy to harder exercise	15–20 min
secondary inpatient cardiac rehabilitation	Respiratory muscle training 7 days/week, (15 min)	7 days/week	From low to moderate intensity, 10 repetitions, rest between sessions.	15 min

**Table 8:** Comparison Between Patients Who Came to Follow-Up and Those Who Did Not

Variable	Follow-Up (*n* = 58)	Drop out (*n* = 33)	*p*-value
Age	72.4 ± 10.2	72.6 ± 5.2	0.916
General EFS Score at V1	6.0 ± 1.8	6.2 ± 1.6	0.760
Gender (M/F)	40 / 18	17 / 16	0.153
Group (Control/Intervention)	25 / 33	19 / 12	0.211

*EFS* Edmonton Frailty Scale

Values are shown as mean ± standard deviation or n (count) for categorical variables.

*P*-values reflect differences between groups using independent samples t-test or chi-square as appropriate

**Table 9:** Edmonton Frailty Scale Category Transitions (Complete Case: V1→V2→V3)

Frailty Category	CG V1 (*n* = 50)	CG V2 (*n* = 45)	CG V3 (*n* = 25)	IG V1 (*n* = 50)	IG V2 (*n* = 46)	IG V3 (*n* = 33)
**Robust (1–3)**	0 (0%)	2 (4%)	2 (8%)	0 (0%)	6 (13%)	7 (21%)
**Prefrail (4–5)**	20 (40%)	22 (49%)	12 (48%)	21 (42%)	25 (54%)	22 (67%)
**Frailty (6–17)**	30 (60%)	21 (47%)	11 (44%)*	29 (58%)	15 (33%)	4 (12%)*

Complete case analysis: CG n=25 of 50 (50% dropout at V3), IG n=33 of 50 (34% dropout)

*Significant improvement IG Frailty V1→V3 (p<0.001);CG Frailty V1→V3 P<0.01).Nosignificant between-group differences were observed (χ² p=0.32)

## Abbreviations

EFS: Edmonton Frail Scale
CR: Cardiac rehabilitation
ET: Exercise training
IG: Intervention group
CG: Control group
TUG: Timed up and go test
MET: Metabolic equivalent
6 MWT: 6 – minute walking test
maxHR: Maximum heart rate
V1: First assessment
V2: Second assessment
V3: Third assessment
